# Joint Modeling of Resistance to Six Antimicrobials in Urinary Escherichia coli Isolates in Quebec, Canada

**DOI:** 10.1128/AAC.02531-18

**Published:** 2019-06-24

**Authors:** Jean-Paul R. Soucy, Alexandra M. Schmidt, Charles Frenette, Patrick Dolcé, Alexandre A. Boudreault, David L. Buckeridge, Caroline Quach

**Affiliations:** a Division of Epidemiology, Dalla Lana School of Public Health, University of Toronto, Toronto, Ontario, Canada; b Department of Epidemiology, Biostatistics and Occupational Health, McGill University, Montréal, Quebec, Canada; c Infectious Disease Department, McGill University Health Centre, Montréal, Quebec, Canada; d Department of Medical Microbiology and Infectious Diseases, Centre Hospitalier Régional de Rimouski, Rimouski, Quebec, Canada; e Département de Microbiologie-infectiologie et d’Immunologie, Université Laval, Québec City, Quebec, Canada; f CHU de Québec, Université Laval, Québec City, Quebec, Canada; g Department of Microbiology, Infectious Diseases & Immunology, Université de Montréal, Montréal, Quebec, Canada; h Infection Prevention & Control Unit, Department of Pediatric Laboratory Medicine, CHU Sainte-Justine, Université de Montréal, Montréal, Quebec, Canada

**Keywords:** *Escherichia coli*, hierarchical modeling, laboratory data, resistance, surveillance

## Abstract

Empirical treatment of urinary tract infections should be based on susceptibility profiles specific to the locale and patient population. Additionally, these susceptibility profiles should account for correlations between resistance to different types of antimicrobials. We used hierarchical logistic regression models to investigate geographic, temporal, and demographic trends in resistance to six antimicrobials in community-acquired and nosocomial urinary E. coli isolates from three communities in the province of Quebec, Canada, procured between April 2010 and December 2017. A total of 74,986 community-acquired (patient age, ≥18 years) and 4,384 nosocomial isolates (patient age, ≥65 years) were analyzed. In both community-acquired and nosocomial isolates, we found geographic variation in the prevalence of resistance. Male sex (community-acquired hierarchical mean odds ratio [OR], 1.24; 95% credible interval [CI], 1.02 to 1.50; nosocomial hierarchical mean OR, 1.16, 95% CI, 0.92 to 1.41) and recent hospitalization (community-acquired hierarchical mean OR, 1.49; 95% CI, 1.33 to 1.66; nosocomial hierarchical mean OR, 1.31; 95% CI, 0.99 to 1.78) were associated with a higher risk of resistance to most types of antimicrobials. We found distinct seasonal trends in both community-acquired and nosocomial isolates, but only community-acquired isolates showed a consistent annual pattern. Ciprofloxacin resistance increased sharply with patient age. We found clinically relevant differences in antimicrobial resistance in urinary E. coli isolates between locales and patient populations in the province of Quebec. These results could help inform empirical treatment decisions for urinary tract infections. In the future, similar models integrating local, provincial, and national resistance data could be incorporated into decision support systems for clinicians.

## Abstract (precis)

Empirical treatment of urinary tract infections should be based on susceptibility profiles specific to the locale and patient population. Additionally, these susceptibility profiles should account for correlations between resistance to different types of antimicrobials.

## INTRODUCTION

Urinary tract infections (UTIs) are among the mostly commonly encountered infections in community and hospital settings (1, 2). By far the most frequently isolated pathogen in these infections is the Gram-negative, facultatively anaerobic bacteria Escherichia coli (2, 3). UTIs are generally treatable with a short course of antibiotics, although the prevalence of uropathogens resistant to one or more antibiotics, such as extended spectrum β-lactamase-producing (ESBL) E. coli, is rising (1, 4–7). Empirical therapy for UTI is often prescribed without a urine culture or before the results of susceptibility testing are available, with 20% being cited as a threshold past which empirical treatment is compromised (8). Significant variation exists between regions (4, 9–12). Consequently, Infectious Diseases Society of America (IDSA) guidelines recommend that treatment selection be based on local or regional resistance rates (8); there is also a need for early identification of patients at risk for treatment-resistant infections.

Much of the previous research on risk factors for antimicrobial resistance in urinary E. coli infections relies on crosstabular methods that present resistance proportions for various subgroups (e.g., men versus women, children versus adults), which makes it difficult to appreciate the relative contributions of each variable to observed variation in the prevalence of resistance between patients, places, times, and settings of acquisition (community-acquired or nosocomial). Additionally, resistance in an isolate to each antimicrobial is typically treated as if it is independent of others, even though mechanisms of antimicrobial resistance are rarely confined to a single drug (13, 14). For example, the genes in ESBL-producing E. coli conferring penicillin and cephalosporin resistance often cooccur on plasmids with genes granting resistance to other classes of antimicrobials, such as aminoglycosides, trimethoprim-sulfamethoxazole, and quinolones (15). Appropriate statistical methods can leverage these correlations to more precisely estimate risk factors for each type of resistance. Advances in data interoperability have made it easier to perform studies integrating a greater breadth of data, rather than relying on relatively small samples collected over a short period of time, often from a single hospital.

In the province of Quebec, Canada, the great majority of microbiological testing, including antimicrobial susceptibility testing, is done in hospital rather than in private laboratories. Adult urinary E. coli isolates are regularly tested for resistance to six antimicrobials representing five classes, ampicillin (penicillin), gentamicin (aminoglycoside), ciprofloxacin (fluoroquinolone), nitrofurantoin (nitrofuran), trimethoprim-sulfamethoxazole (TMP-SMX; combination dihydrofolate reductase inhibitor/sulfonamide), and tobramycin (aminoglycoside). Several major health care networks have adopted a common infection control software (Nosokos; Nosotech, Rimouski, Canada), facilitating the aggregation of many years of susceptibility testing results from across the province using a common data dictionary. Using hierarchical logistic regression methods, it is possible to overcome the limitations of previous research by simultaneously analyzing many potential risk factors and allowing risk factors to be correlated across multiple types of resistance to different antimicrobials. The latter also permits the estimation of an overall association for a risk factor across all types of resistance. In this study, we developed models to investigate the temporal, geographic, and patient-level predictors of resistance to six antimicrobials in a large sample of community-acquired and nosocomial urinary E. coli isolates from three communities in the province of Quebec, Canada.

## RESULTS

### Community-acquired isolates.

**FIG 1:**
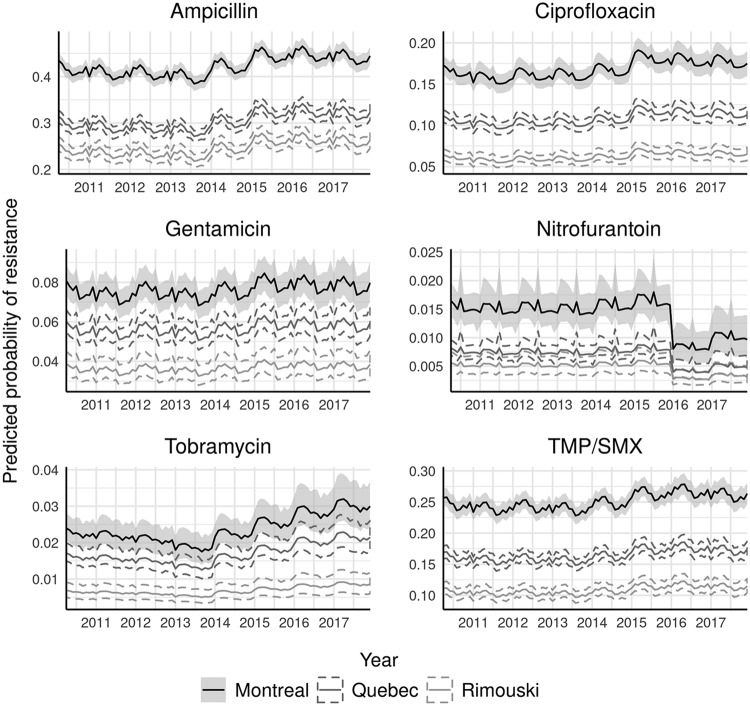
Posterior summary of monthly probability of resistance to six antimicrobials (with 95% credible intervals) for community-acquired urinary Escherichia coli isolates (*n* = 74,986) from three communities in Quebec, Canada, between April 2010 and December 2017. Probabilities calculated for isolates taken from women approximately 55 years of age who were not hospitalized in the past 30 days. Credible intervals represented by shading for Montreal and dashed lines for other communities. Each antimicrobial is plotted using a scale proportional to its baseline prevalence of resistance.

Similar annual and seasonal patterns were observed across most types of resistance in community-acquired isolates (Fig. 1). Compared to 2010, the odds of resistance held steady or slightly declined between 2011 and 2014, after which resistance increased from 2015 to 2017. The exceptions to this pattern were gentamicin, resistance to which remained relatively constant throughout the study period, and nitrofurantoin, resistance to which declined in 2016 and 2017. A seasonal trend was observed, with resistance generally peaking between February and May compared to that in January. Resistance differed strongly by geography, with Montreal generally having the highest prevalence of resistance and Rimouski the lowest (Fig. 1).

**FIG 2:**
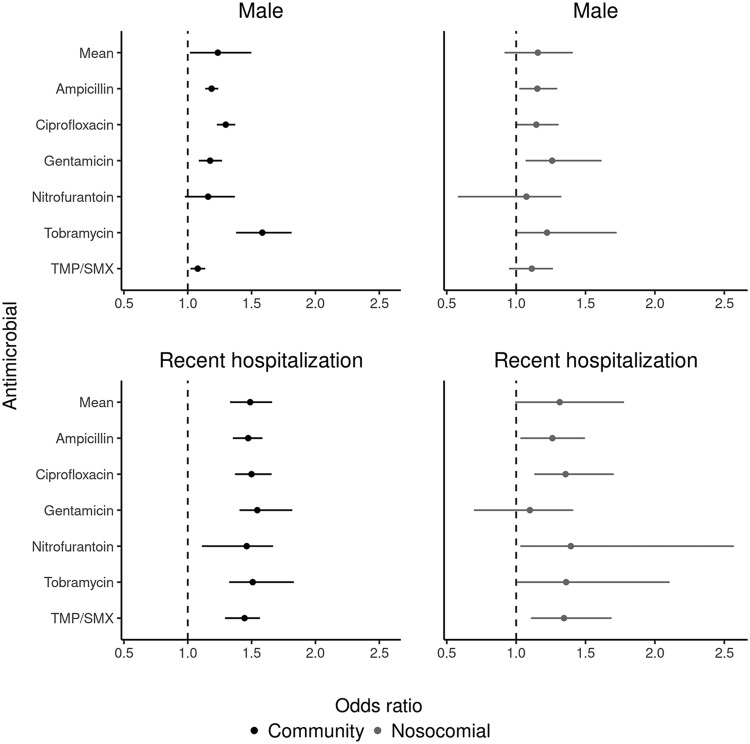
Odds ratios (with 95% credible intervals) for male sex and recent hospitalization for resistance to six antimicrobials in community-acquired and nosocomial urinary Escherichia coli isolates (*n* = 79,370) from three communities in Quebec, Canada, between April 2010 and December 2017. Estimated mean association (from the hierarchical model) across all antimicrobials is also shown. The null (OR = 1) is denoted with a dashed line.

**FIG 3:**
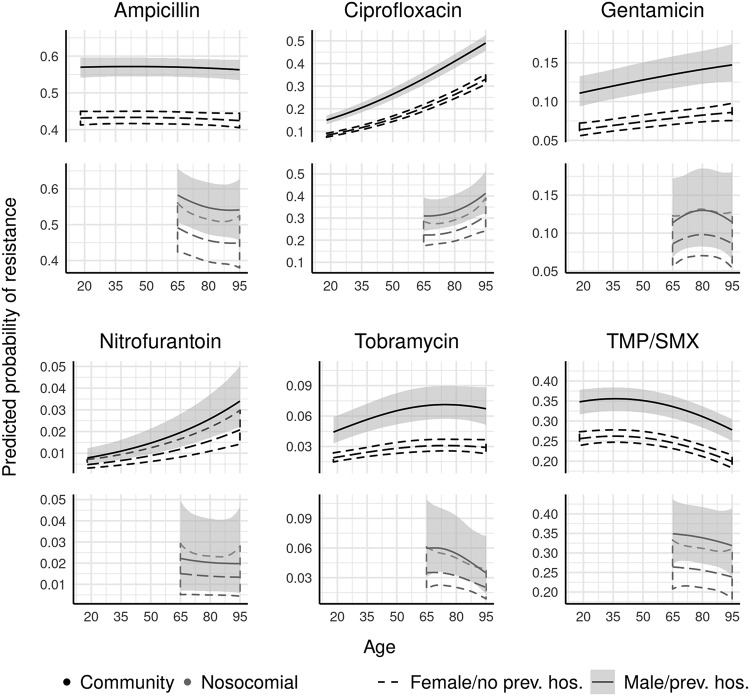
Posterior summary of probability of resistance to six antimicrobials (with 95% credible intervals) for community-acquired (*n* = 74,986) and nosocomial (*n* = 4,384) urinary Escherichia coli isolates, comparing isolates from women who were not hospitalized in the past 30 days to isolates from men who were hospitalized in the past 30 days. Probabilities calculated for isolates taken from patients in Montreal during January of 2017. Credible intervals represented by dashed lines for the first patient group and shading for the second patient group. Each antimicrobial is plotted using a scale proportional to its baseline prevalence of resistance.

Male sex (hierarchical mean odds ratio [OR], 1.24; 95% credible interval [CI], 1.02 to 1.50) and hospitalization in the past 30 days (hierarchical mean OR, 1.49; 95% CI, 1.33 to 1.66) were consistent risk factors across all types of resistance (Fig. 2). Age was strongly associated with increased resistance to ciprofloxacin (Fig. 3). Exact values for model parameters on the odds ratio scale are given in Table S1.

### Nosocomial isolates.

**FIG 4:**
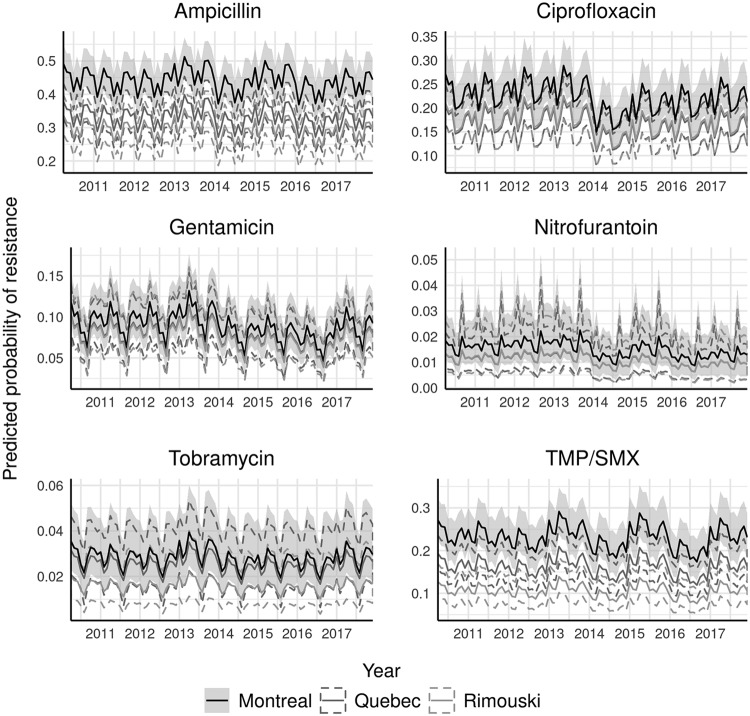
Posterior summary of monthly probability of resistance to six antimicrobials (with 95% credible intervals) for nosocomial urinary Escherichia coli isolates (*n* = 4,384) from three communities in Quebec, Canada between April 2010 and December 2017. Probabilities calculated for isolates taken from women approximately 80 years of age not hospitalized in the past 30 days. Credible intervals represented by shading for Montreal and dashed lines for other communities. Each antimicrobial is plotted using a scale proportional to its baseline prevalence of resistance.

Nosocomial isolates lacked a clear annual trend (Fig. 4). There was a weak tendency for resistance to be lower in February and July through September compared to that in January (Fig. 4). There was a tendency for Montreal to have a higher prevalence of resistance than the other two cities, particularly for ampicillin, ciprofloxacin, and TMP-SMX (Fig. 4).

Male sex was significantly (credible interval does not cross 1, the null) positively associated with resistance to three antimicrobials, and trended positively in the rest (hierarchical mean OR, 1.16; 95% CI, 0.92 to 1.41) (Fig. 2). Recent prior hospitalization was a risk factor (hierarchical mean OR, 1.31; 95% CI, 0.99 to 1.78) (Fig. 2). Age was again strongly associated with increased ciprofloxacin resistance (Fig. 3). Exact values for model parameters on the odds ratio scale are given in Table S2.

## DISCUSSION

Empirical treatment of urinary tract infections should be based on susceptibility profiles specific to the locale and patient population. Susceptibility testing data capable of informing treatment decisions is present in hospital information systems, but has to date been underutilized for this purpose, due to a lack of appropriate statistical methods and interoperability across systems. In this study, we jointly modelled the temporal, geographic, and patient-level associations with resistance to six antimicrobials in urinary E. coli isolates from inpatients and outpatients from three cities across the province of Quebec using a shared data structure.

Our results showed commonalities in risk factors across drugs and between community-acquired and nosocomial isolates. The marked divergence in the prevalence of resistance between the three cities in our study underscores the importance making local resistance data available to prescribers, rather than relying on provincial or national estimates. Men and recently hospitalized individuals were generally at a higher risk for antimicrobial resistance, whereas age predicted higher resistance to only some antimicrobials. Our hierarchical approach accounted for correlations between types of resistance and enabled us to more precisely estimate risk factors for individual types of resistance in specific patient populations. As an illustration, consider a frontline clinician in Quebec City who uses a local antibiogram to determine that the average rate of susceptibility to TMP-SMX in urinary E. coli is 82%. The clinician also knows that sex and hospitalization in the past 30 days influence susceptibility rates, but does not know the precise magnitude of these influences. With the type of model we fit in this study, the clinician would know to expect 84.2% (95% CI, 83.1% to 85.2%) susceptibility if the patient were a 70-year-old woman but only 75.2% (95% CI, 72.9% to 77.6%) susceptibility if the patient were a 30-year-old, recently hospitalized woman. These two predicted susceptibilities are on opposite sides of the 80% susceptibility threshold cited for empirical treatment with TMP-SMX (8).

This study’s results support some of the findings from previous research. Both male sex and recent hospitalization have been previously identified as positive predictors of resistance in urinary E. coli (9, 16–20), although the association with sex is not uniform across drugs (e.g., see references 9 and 19). While male sex typically accounted for only a few percentage points of increased risk in our study, when combined with stronger risk factors, such as recent hospitalization, community, and potentially age, dramatically different resistance profiles can result, particularly for the mainline treatment TMP-SMX (e.g., see Fig. 1 and 3). Differences in etiology between men and women may be important, as community-acquired UTIs are common in otherwise healthy women (21) but in men are often associated with anatomical abnormalities, such as an enlarged prostate (22). Nonetheless, the tendency for men to have higher resistance to some antimicrobials persisted in the nosocomial setting, despite the similarity in the primary route of acquisition (urinary catheterization). Rising rates of resistance over time are consistent with trends in urinary E. coli (23) and pathogens in general (24).

The evidence regarding the relationship between age and resistance in urinary E. coli suggests that the association is highly dependent on the drug in question (9, 19, 20). Age has been consistently linked to an elevated risk of ciprofloxacin resistance in adults (4, 19, 20, 25, 26), as observed in our fitted model. This may be linked to cumulative changes in the gut microbiome resulting from the use of broad-spectrum antimicrobials like ciprofloxacin (27), altering an individual’s susceptibility to invasion by antimicrobial-resistant organisms (28). Seasonal trends in community-acquired infections likely relate to patterns of antibiotic prescribing (29, 30) and may also be influenced by other factors, like international travel (31). Resistance rates were remarkably higher in densely urban Montreal compared to the other two sites, with the more remote Rimouski generally showing the lowest rates. Our results are consistent with the hypothesized positive association between population density and resistance (32), although we cannot draw general conclusions from only three sites. It should be noted, however, that the McGill University Health Centre serves as a major reference center for urology in Montreal and may receive complex cases from other hospitals, which could be overrepresenting the prevalence of resistance in the city.

Surprisingly, our model does not generally predict large differences in the risk of antimicrobial resistance among isolates from elderly (65+ years of age) individuals based on setting of acquisition (community or nosocomial; see Fig. 3). A metaanalysis of 54 observational studies by Fasugba et al. (23) concluded that ciprofloxacin resistance was higher in hospital-acquired (38%; 95% confidence interval, 36% to 41%) compared with community-acquired (27%; 95% confidence interval, 24% to 31%) infections, although they did not directly control for confounders such as sex, previous hospitalization, or age among adult patients. Fleming et al. (33) reported that in 156 urinary E. coli taken from a Georgia hospital, prevalence of resistance to TMP-SMX (in addition to that to several other antimicrobials) was higher in the hospital-acquired (34.6%) than the community-acquired (25.2%) isolates, but did not adjust for demographic factors. Other studies (4, 34, 35) also report differences in rates of resistance between these two subpopulations (interestingly, Lob et al. [4] report these differences in the United States but not Canada), but it is also necessary to note the wide variation in estimated rates of resistance between studies. Geographic differences, as well as the demographic associations detected in our large database of isolates, help to explain this variation. It is possible that, had we compared nosocomial isolates from adults under 65 (which are much less common) to community-acquired isolates in the same age range, the results might have been different.

Our study had several limitations. The lack of supplementary clinical data (e.g., symptoms and clinical outcomes) meant that we were unable to distinguish asymptomatic bacteriuria from symptomatic UTI, or the type and severity of infections; we also lacked data on many factors potentially relevant to treatment, such as catheterization, prior antibiotic use, comorbid illnesses, and drug interactions. Since our sampling frame included only patients for which clinical specimens had been taken, and specimens may not be systematically taken for uncomplicated UTIs, our data set may not be reflective of all treated UTIs. The purpose of a standard antibiogram is to provide a baseline expected level of resistance, and our model improves these baseline predictions by making them more precisely targeted to particular patient groups. These models are intended to supplement, not replace, good clinical judgment, especially regarding the usually unnecessary treatment of asymptomatic bacteriuria and the judicious use of broad-spectrum antimicrobials.

Changes in clinician behavior over time or between locations could explain some of the variability in rates of resistance. Since we cannot confirm the length of catheter use in hospitalized patients or identify when a patient was hospitalized in a hospital network outside where their sample was tested (more likely in Montreal and Quebec City, which have many hospitals), there is a possibility of misclassifying nosocomial isolates as community-acquired, as well as of incorrectly identifying previous hospitalization status. This could obscure the differences we observed between nosocomial and community-acquired isolates and blunt the increased risk of resistance seen in recently hospitalized patients.

The value of electronic health records for surveillance, quality improvement, and epidemiological research is increasing as databases become more interlinked. This study demonstrates the utility of standardized antimicrobial resistance data from multiple institutions to produce locally relevant profiles of antimicrobial resistance. Our modeling approach allowed us to make inferences about temporal, geographic, and demographic variation in the probability of resistance to 6 types of antimicrobials used in the treatment of urinary tract infections. Clinically relevant differences in resistance between communities and patient populations in the province of Quebec could inform empirical treatment decisions. In the future, a model-based approach for antimicrobial resistance informed by local, provincial, and national trends could be incorporated into decision support systems for clinicians.

## MATERIALS AND METHODS

### Study population.

All urine cultures positive for E. coli tested for antimicrobial susceptibility in four hospital laboratories in three cities across the province of Quebec, Canada, between April 2010 and December 2017 were included. The sampled communities and laboratories were from Montreal (large urban; McGill University Health Centre), Quebec City (smaller urban; Centre hospitalier universitaire de Québec, Centre hospitalier affilié universitaire de Québec), and Rimouski (small, remote; Hôpital régional de Rimouski). For each isolate, the following data were available: date of collection, age, sex, and unique patient identifier, date of previous admission/localization in the hospital (e.g., emergency room), and antimicrobial testing results classified as sensitive, intermediate, or resistant. Species identification was performed according to local procedures and susceptibility testing was generally done using a Vitek 2 system following CLSI breakpoints and guidelines (36). Samples taken at least 48 h after hospital admission or within 48 h after discharge were classified as nosocomial (based on National Healthcare Safety Network guidelines [37]); other samples (e.g., from emergency departments, outpatient clinics, and community clinics) were classified as community acquired. The characteristics of the study population are summarized in Table 1.

**TABLE 1:** Characteristics of 79,370 urinary E. coli isolates collected from three communities in Quebec, Canada between April 2010 and December 2017

Patient characteristic	Infection type (no. [%] of patients)	
	Community-acquired	Nosocomial
All	74,986	4,384
Sex		
Male	11,079 (14.8%)	1,310 (29.9%)
Female	63,907 (85.2%)	3,074 (70.1%)
Age category		
18–64	44,981 (60.0%)	
65–95	30,005 (40.0%)	4,384
Prior hospitalization in the past 30 days	1,743 (2.3%)	392 (8.9%)
Community		
Montreal	16,396 (21.9%)	1,478 (33.7%)
Quebec City	51,942 (69.3%)	2,296 (52.4%)
Rimouski	6,648 (8.9%)	610 (13.9%)
Resistance		
Ampicillin	24,713 (33.0%)	1,683 (38.4%)
Ciprofloxacin	9,930 (13.2%)	896 (20.4%)
Gentamicin	4,572 (6.1%)	375 (8.6%)
Nitrofurantoin	719 (1.0%)	60 (1.4%)
Tobramycin	1,340 (1.8%)	120 (2.7%)
TMP-SMX	13,037 (17.4%)	819 (18.7%)

### Selection criteria.

The initial database contained 163,541 isolates identified as E. coli. We excluded samples with more than two bacterial species (considered contaminated according to the CDC [https://www.cdc.gov/nhsn/faqs/faq-uti.html]) and randomly retained one isolate when a sample contained more than one isolate of the same species (as distinguished by morphology and/or susceptibility patterns). We excluded isolates with no susceptibility testing results or patient sex available. We restricted to patients aged between 18 and 95 years, as results for ciprofloxacin resistance were regularly suppressed for children (38) and because we wanted to avoid unwarranted extrapolation at very high ages (which were rare and included a few unrealistic values). We restricted nosocomial isolates to those obtained from patients aged 65 years and older (70% of isolates) to avoid extrapolation to age groups underrepresented in our sample. We excluded isolates without results for all six antimicrobials of interest, leaving 111,153 isolates. We assumed statistical independence across individuals and retained only the first isolate from each of the 79,370 unique patients (71% unique). The exclusion flowchart is given in Fig. S1.

### Outcomes.

We included the following six binary outcomes: susceptibility (0) or resistance (1) to ampicillin, gentamicin, ciprofloxacin, nitrofurantoin, TMP-SMX, and tobramycin. The small percentage of susceptibility results classified as intermediate (1.7%) were classified as susceptible for the purpose of analysis (“nonresistant” isolates; e.g., see references 29, 39, 40).

### Covariates.

Covariates included in the analysis were community (Quebec City and Rimouski compared to Montreal), sex (male compared to female), age (quadratic), hospitalization in the past 30 days (yes compared to no), month (compared to January), and year (compared to 2010).

### Analysis.

We fitted two independent but identical models, one for community-acquired isolates (*n* = 74,986) and one for nosocomial isolates (*n* = 4,384). We jointly modelled six binary outcomes (the presence or absence of resistance to each of the six tested antibiotics) using multivariate hierarchical logistic regression in a Bayesian framework (for an overview of this approach, see Gelman and Hill [41]). Briefly, this approach gives each outcome (type of resistance) its own intercept and set of regression coefficients while explicitly accounting for nonindependence between the outcomes by assuming these parameters arise from a common distribution; this accounts for the fact that resistance to one type of antibiotic may be related to resistance to other types of antibiotics. With this technique, the precision of regression coefficients for risk factors for each type of resistance is improved by using all available information, to the extent supported by the data, rather than incorrectly treating each type of resistance as independent. This method also allows us to estimate a “hierarchical mean,” which serves as an overall estimate of the association with the risk factor across all types of resistance.

Inference is performed under the Bayesian paradigm with diffuse prior distributions assigned to the unknowns in the model. We used Markov chain Monte Carlo (MCMC), as implemented in the nimble (42) package (version 0.6-10) in R version 3.4.4 (43), to generate samples from the posterior distribution of model parameters. The 95% posterior credible intervals (CI) for each parameter are presented after 10% burn-in of MCMC samples. A full model description, with details on model fitting, is available in the supplemental methods; example code with simulated data is available as supplemental material.

### Ethics.

We obtained ethics approval from each participating institution according to the Multi-Centre Research Ethics Review Mechanism of the Quebec Ministry of Health and Social Services (MP-37-2018-3758).

## Supplementary Material

Supplemental file 1
