# Supplementary material for: Joint Modeling of Resistance to Six Antimicrobials in Urinary Escherichia coli Isolates in Quebec, Canada
Source: Antimicrob Agents Chemother. 2019 Jun 24;63(7):e02531-18. doi: 10.1128/AAC.02531-18 (PMC6591649; doi:10.1128/AAC.02531-18)
Supplement: Supplemental file 1 [file AAC.02531-18-s0001.pdf]

Supplementary Methods

Detailed model description

We fit two independent but identical models: one for community-acquired isolates (n = 74,986) and one for nosocomial isolates (n = 4,384). We jointly modelled six binary outcomes (let $Y_{i,a}$ be the presence or absence of resistance to antimicrobial *a* in isolate *i*) using hierarchical logistic regression in a Bayesian framework (for an overview of this approach, see Gelman and Hill (1)). Each outcome had its own intercept and set of regression coefficients $\beta_{p,a}$ describing the association between predictor *p* and the log odds of resistance to antimicrobial *a*. The hierarchical (multilevel) structure of the model imposes a correlation structure on the outcomes by assuming that coefficients $\beta_{p,a}$ for a particular covariate *p* and associated with antibiotic *a* are normally distributed around a common mean for that covariate (β_p) with standard deviation σ_p. By allowing the association between, for example, male sex and the probability of resistance to be related between different types of antimicrobial resistance, the precision of coefficient estimates is improved through the "borrowing of strength" across outcomes. This hierarchical mean β_p also serves as an overall estimate of the association with the risk factor across all types of resistance. The model is as follows:

$$Y_{i,a} \mid \pi_{i,a} \sim \text{Bernoulli}(\pi_{i,a})$$
$$\text{logit}(\pi_{i,a}) = \beta_{p,a} X_i \qquad \beta_{p,a} \sim N(\beta_p, \sigma_p^2).$$

Covariate values for each isolate *i* are given as a *p*-dimensional vector X_i. Each common mean β_p was assigned a diffuse normal prior (mean = 0, variance = 10,000); each standard deviation σ_p was assigned a diffuse half-Cauchy prior (scale = 25) (2). Samples from the resultant posterior distribution of the parameters were obtained using Markov chain Monte Carlo (MCMC) methods as implemented in the nimble (3) package (version 0.6-10) in R 3.4.4 (4). The 95% posterior credible intervals for each parameter are presented after 10% burn-in of MCMC samples.

Markov chain Monte Carlo methods

For both datasets, age was centred at its mean value prior to modelling. For community-acquired isolates, this was 55.2; for nosocomial isolates, this was 79.7. All parameters were assigned diffuse normal prior distributions (mean = 0, variance = 10,000), with the exception of the multilevel standard deviation parameters, which were assigned a diffuse half-Cauchy prior distribution (scale = 25). Models were run for 30,000 iterations, discarding the first 10% as burn-in.

Residuals were assessed using binned residual plots as described by Gelman & Hill (5). Chains were visually inspected for convergence. Finally, a second chain was run using different initial values and results were compared to verify convergence had actually occurred.

Example code with simulated data is available as supplementary material below.

References

1. Gelman A, Hill J. 2006. Data Analysis Using Regression and Multilevel/Hierarchical Models. Cambridge University Press, Cambridge.

2. Gelman A. 2006. Prior distributions for variance parameters in hierarchical models (Comment on Article by Browne and Draper). Bayesian Anal 1:515–534.

3. de Valpine P, Turek D, Paciorek CJ, Anderson-Bergman C, Lang DT, Bodik R. 2017. Programming With Models: Writing Statistical Algorithms for General Model Structures With NIMBLE. J Comput Graph Stat 26:403–413.

4. R Core Team. 2018. R: A Language and Environment for Statistical Computing. R Foundation for Statistical Computing, Vienna, Austria.

5. Gelman A, Hill J. 2006. Logistic regression, p. 79–108. *In* Data Analysis Using Regression and Multilevel/Hierarchical Models. Cambridge University Press, Cambridge.

Figure S1. Flowchart of exclusion criteria for *Escherichia coli* isolates included in the study.

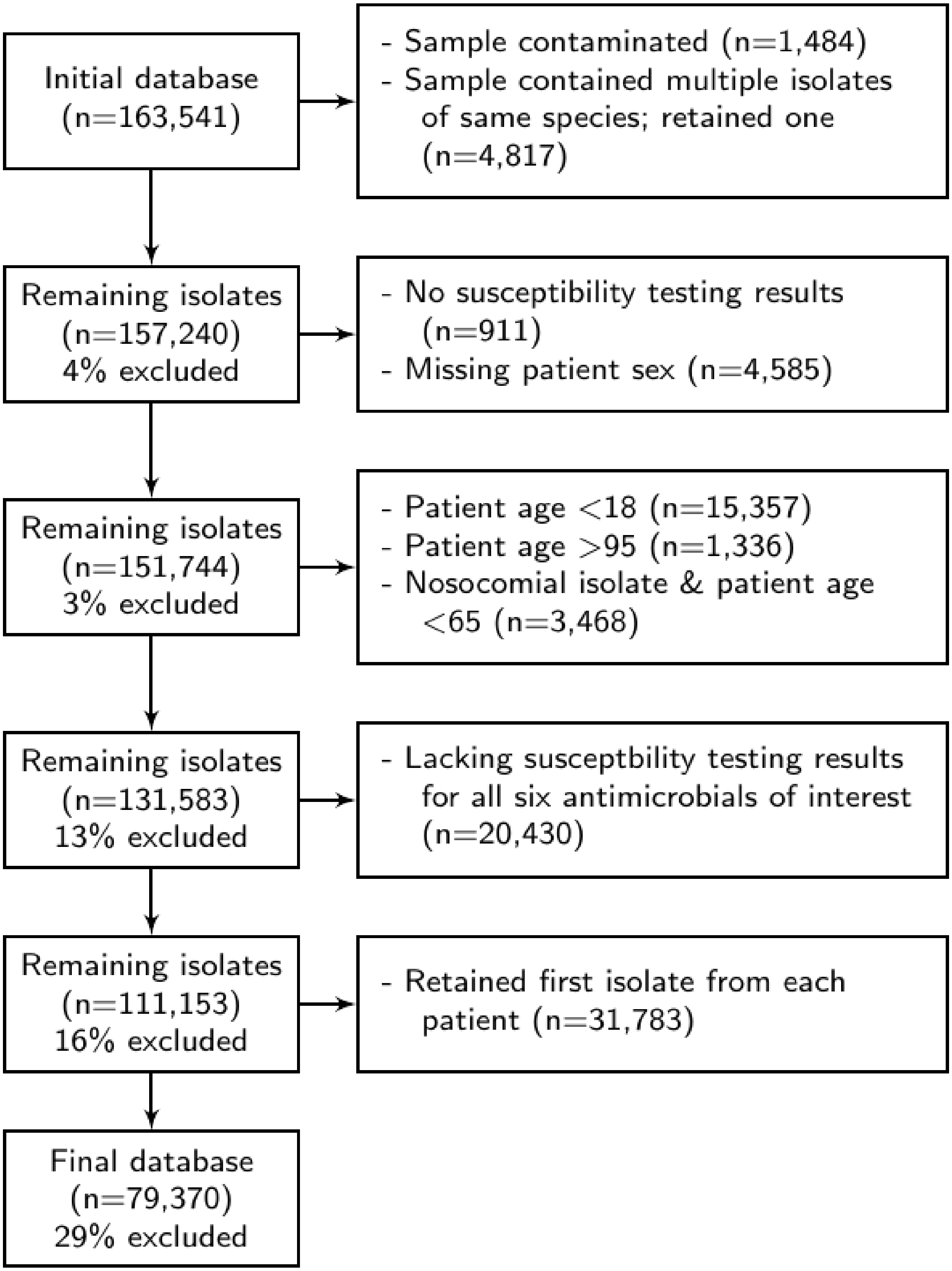

Table S1. Odds ratios (with 95% credible intervals) for all variables in the hierarchical multivariate logistic regression model for resistance to six antimicrobials in community-acquired urinary *Escherichia coli* isolates (n = 74,986).

**Coefficient**	**Hierarchical mean**	**Ampicillin**	**Ciprofloxacin**	**Gentamicin**	**Nitrofurantoin**	**Tobramycin**	**TMP/SMX**
Intercept*	0.10 (0.02, 0.65)	0.69 (0.65, 0.74)	0.19 (0.18, 0.21)	0.08 (0.07, 0.09)	0.02 (0.01, 0.02)	0.02 (0.02, 0.03)	0.32 (0.29, 0.34)
Sex (male)	1.24 (1.02, 1.50)	1.19 (1.14, 1.24)	1.30 (1.23, 1.37)	1.18 (1.09, 1.27)	1.16 (0.98, 1.37)	1.58 (1.38, 1.81)	1.08 (1.02, 1.14)
Age (10 years)	1.08 (0.95, 1.24)	1.00 (0.99, 1.00)	1.25 (1.23, 1.26)	1.04 (1.03, 1.06)	1.21 (1.17, 1.26)	1.06 (1.04, 1.09)	0.96 (0.95, 0.97)
Age (10 years)^2	1.00 (0.98, 1.00)	1.00 (1.00, 1.00)	1.00 (0.99, 1.00)	1.00 (0.99, 1.00)	1.00 (0.99, 1.01)	0.98 (0.97, 1.00)	0.99 (0.99, 0.99)
Hosp. past 30 days	1.49 (1.33, 1.66)	1.47 (1.35, 1.59)	1.50 (1.37, 1.66)	1.54 (1.41, 1.82)	1.46 (1.11, 1.67)	1.51 (1.32, 1.83)	1.44 (1.29, 1.56)
Quebec	0.61 (0.50, 0.74)	0.60 (0.58, 0.62)	0.60 (0.57, 0.63)	0.73 (0.68, 0.78)	0.49 (0.42, 0.57)	0.71 (0.63, 0.79)	0.59 (0.57, 0.61)
Rimouski	0.37 (0.27, 0.47)	0.45 (0.42, 0.48)	0.33 (0.30, 0.36)	0.46 (0.40, 0.53)	0.34 (0.24, 0.44)	0.28 (0.20, 0.37)	0.36 (0.33, 0.39)
2011	0.96 (0.90, 1.03)	0.96 (0.91, 1.02)	0.93 (0.86, 1.00)	0.96 (0.88, 1.04)	0.96 (0.88, 1.12)	0.96 (0.89, 1.09)	0.96 (0.90, 1.03)
2012	0.97 (0.89, 1.04)	0.96 (0.91, 1.01)	0.97 (0.91, 1.03)	0.99 (0.93, 1.10)	0.98 (0.89, 1.13)	0.94 (0.76, 1.01)	0.98 (0.92, 1.04)
2013	0.93 (0.81, 1.04)	0.93 (0.88, 0.98)	0.96 (0.90, 1.04)	0.95 (0.87, 1.04)	0.95 (0.83, 1.15)	0.82 (0.65, 0.96)	0.95 (0.90, 1.02)
2014	1.00 (0.94, 1.07)	1.02 (0.97, 1.08)	1.00 (0.94, 1.06)	1.00 (0.91, 1.06)	1.01 (0.93, 1.13)	1.00 (0.87, 1.07)	1.01 (0.95, 1.07)
2015	1.10 (1.00, 1.19)	1.12 (1.07, 1.19)	1.12 (1.05, 1.20)	1.06 (0.95, 1.14)	1.08 (0.90, 1.18)	1.12 (1.02, 1.29)	1.09 (1.02, 1.16)
2016	1.00 (0.63, 1.53)	1.13 (1.06, 1.21)	1.09 (1.01, 1.19)	1.04 (0.93, 1.16)	0.53 (0.38, 0.74)	1.26 (1.06, 1.49)	1.11 (1.03, 1.20)
2017	1.03 (0.73, 1.41)	1.10 (1.03, 1.18)	1.08 (0.99, 1.18)	1.03 (0.92, 1.16)	0.66 (0.45, 0.92)	1.34 (1.11, 1.61)	1.08 (1.00, 1.17)
Feb	1.08 (1.01, 1.17)	1.08 (1.03, 1.15)	1.08 (1.01, 1.15)	1.08 (1.00, 1.17)	1.10 (1.01, 1.34)	1.08 (0.97, 1.20)	1.07 (0.99, 1.13)
Mar	1.08 (1.01, 1.18)	1.07 (1.01, 1.13)	1.10 (1.03, 1.19)	1.07 (0.99, 1.17)	1.09 (1.00, 1.32)	1.09 (0.99, 1.24)	1.05 (0.97, 1.12)
Apr	1.10 (1.03, 1.17)	1.11 (1.05, 1.17)	1.09 (1.01, 1.15)	1.11 (1.04, 1.20)	1.10 (1.01, 1.23)	1.10 (1.01, 1.20)	1.09 (1.02, 1.15)
May	1.07 (1.00, 1.14)	1.08 (1.01, 1.14)	1.07 (0.99, 1.13)	1.07 (0.99, 1.14)	1.07 (0.95, 1.16)	1.08 (0.98, 1.17)	1.09 (1.02, 1.17)
Jun	1.03 (0.95, 1.09)	1.02 (0.96, 1.08)	1.03 (0.96, 1.10)	1.03 (0.96, 1.12)	1.01 (0.84, 1.09)	1.03 (0.93, 1.13)	1.04 (0.98, 1.10)
Jul	1.05 (0.97, 1.20)	1.02 (0.95, 1.08)	1.06 (0.99, 1.15)	1.07 (0.98, 1.18)	1.12 (0.99, 1.52)	1.03 (0.90, 1.18)	1.03 (0.96, 1.11)
Aug	0.98 (0.92, 1.07)	0.97 (0.92, 1.03)	0.99 (0.93, 1.07)	0.97 (0.89, 1.05)	0.98 (0.86, 1.11)	1.00 (0.93, 1.21)	0.97 (0.91, 1.03)
Sep	0.98 (0.93, 1.04)	0.98 (0.93, 1.03)	0.99 (0.93, 1.06)	0.98 (0.92, 1.05)	0.99 (0.90, 1.10)	0.98 (0.87, 1.06)	0.98 (0.93, 1.04)
Oct	1.00 (0.94, 1.07)	1.00 (0.94, 1.05)	0.99 (0.92, 1.05)	1.00 (0.93, 1.08)	1.00 (0.90, 1.09)	1.01 (0.93, 1.13)	1.02 (0.96, 1.09)
Nov	1.00 (0.94, 1.07)	1.00 (0.94, 1.05)	1.00 (0.94, 1.08)	1.00 (0.93, 1.07)	1.00 (0.92, 1.12)	1.00 (0.90, 1.08)	1.00 (0.94, 1.06)
Dec	1.04 (0.92, 1.11)	1.05 (0.99, 1.11)	1.03 (0.94, 1.09)	1.06 (0.98, 1.17)	1.01 (0.75, 1.10)	1.03 (0.89, 1.14)	1.05 (0.98, 1.12)

* The intercept refers to a female 55.2 years of age and not hospitalized in the past 30 days in Montreal during January of 2010.

Table S2. Odds ratios (with 95% credible intervals) for all variables in the hierarchical multivariate logistic regression model for resistance to six antimicrobials in nosocomial urinary *Escherichia. coli* isolates (n = 4,384).

**Coefficient**	**Hierarchical mean**	**Ampicillin**	**Ciprofloxacin**	**Gentamicin**	**Nitrofurantoin**	**Tobramycin**	**TMP/SMX**
Intercept*	0.14 (0.02, 1.00)	0.89 (0.70, 1.11)	0.33 (0.25, 0.42)	0.12 (0.08, 0.16)	0.02 (0.01, 0.03)	0.03 (0.02, 0.05)	0.34 (0.26, 0.42)
Sex (male)	1.16 (0.92, 1.41)	1.15 (1.02, 1.30)	1.15 (0.99, 1.31)	1.26 (1.07, 1.62)	1.07 (0.58, 1.33)	1.22 (1.01, 1.72)	1.11 (0.95, 1.26)
Age (10 years)	0.97 (0.80, 1.15)	0.94 (0.87, 1.02)	1.16 (1.05, 1.27)	1.00 (0.89, 1.14)	0.96 (0.75, 1.21)	0.83 (0.64, 1.02)	0.96 (0.87, 1.05)
Age (10 years)^2	0.99 (0.85, 1.12)	1.03 (0.94, 1.12)	1.05 (0.96, 1.18)	0.95 (0.79, 1.07)	1.01 (0.82, 1.29)	0.94 (0.68, 1.08)	0.99 (0.89, 1.09)
Hosp. past 30 days	1.31 (0.99, 1.78)	1.26 (1.03, 1.50)	1.36 (1.13, 1.70)	1.10 (0.70, 1.41)	1.39 (1.03, 2.57)	1.36 (1.01, 2.11)	1.34 (1.11, 1.69)
Quebec	0.73 (0.57, 0.97)	0.63 (0.55, 0.72)	0.69 (0.59, 0.80)	0.86 (0.68, 1.09)	0.72 (0.50, 1.04)	0.88 (0.66, 1.30)	0.65 (0.55, 0.75)
Rimouski	0.60 (0.38, 0.97)	0.53 (0.43, 0.64)	0.72 (0.57, 0.90)	0.83 (0.60, 1.17)	0.72 (0.41, 1.37)	0.55 (0.31, 0.91)	0.39 (0.29, 0.52)
2011	0.98 (0.81, 1.23)	0.95 (0.77, 1.16)	1.01 (0.84, 1.28)	0.99 (0.81, 1.29)	1.00 (0.77, 1.48)	0.96 (0.67, 1.25)	0.98 (0.81, 1.23)
2012	0.98 (0.77, 1.29)	0.93 (0.77, 1.11)	1.07 (0.87, 1.38)	0.99 (0.78, 1.30)	1.07 (0.80, 1.96)	0.93 (0.60, 1.24)	0.91 (0.72, 1.11)
2013	1.11 (0.94, 1.34)	1.09 (0.92, 1.30)	1.10 (0.91, 1.32)	1.12 (0.94, 1.44)	1.12 (0.88, 1.56)	1.11 (0.88, 1.45)	1.11 (0.93, 1.35)
2014	0.81 (0.59, 1.05)	0.87 (0.72, 1.07)	0.75 (0.56, 0.94)	0.84 (0.64, 1.12)	0.77 (0.32, 1.02)	0.86 (0.64, 1.34)	0.83 (0.67, 1.05)
2015	0.96 (0.72, 1.27)	1.03 (0.86, 1.28)	0.91 (0.70, 1.13)	0.86 (0.59, 1.13)	0.99 (0.66, 1.60)	0.92 (0.58, 1.27)	1.08 (0.87, 1.42)
2016	0.81 (0.57, 1.05)	0.86 (0.71, 1.07)	0.89 (0.72, 1.15)	0.74 (0.51, 0.94)	0.75 (0.31, 1.02)	0.86 (0.61, 1.34)	0.78 (0.61, 0.98)
2017	0.95 (0.68, 1.21)	0.94 (0.78, 1.15)	0.96 (0.78, 1.20)	0.94 (0.71, 1.20)	0.87 (0.29, 1.14)	0.99 (0.73, 1.50)	1.02 (0.84, 1.33)
Feb	0.82 (0.60, 1.26)	0.77 (0.59, 0.97)	0.73 (0.53, 0.94)	0.83 (0.61, 1.21)	0.95 (0.65, 2.46)	0.79 (0.46, 1.21)	0.85 (0.66, 1.16)
Mar	0.87 (0.67, 1.09)	0.89 (0.71, 1.12)	0.88 (0.68, 1.11)	0.88 (0.65, 1.17)	0.85 (0.55, 1.19)	0.86 (0.59, 1.17)	0.84 (0.64, 1.07)
Apr	1.12 (0.90, 1.38)	1.09 (0.87, 1.33)	1.13 (0.92, 1.41)	1.16 (0.92, 1.54)	1.11 (0.78, 1.49)	1.11 (0.81, 1.47)	1.11 (0.88, 1.37)
May	1.00 (0.80, 1.24)	0.99 (0.79, 1.20)	1.01 (0.80, 1.26)	0.98 (0.73, 1.23)	1.00 (0.72, 1.37)	1.02 (0.76, 1.41)	1.03 (0.82, 1.31)
Jun	1.02 (0.80, 1.27)	0.98 (0.77, 1.20)	1.05 (0.85, 1.34)	1.03 (0.79, 1.34)	1.02 (0.70, 1.40)	1.00 (0.69, 1.33)	1.02 (0.81, 1.29)
Jul	0.78 (0.61, 0.99)	0.78 (0.63, 0.96)	0.75 (0.58, 0.95)	0.76 (0.55, 0.98)	0.77 (0.53, 1.11)	0.78 (0.55, 1.09)	0.83 (0.66, 1.11)
Aug	0.79 (0.48, 1.08)	0.92 (0.73, 1.20)	0.78 (0.58, 1.00)	0.77 (0.51, 1.05)	0.76 (0.33, 1.17)	0.69 (0.26, 0.99)	0.86 (0.66, 1.14)
Sep	0.81 (0.53, 1.37)	0.79 (0.62, 1.02)	0.82 (0.62, 1.08)	0.58 (0.32, 0.86)	1.18 (0.67, 2.81)	0.88 (0.53, 1.55)	0.80 (0.60, 1.07)
Oct	0.98 (0.77, 1.23)	1.03 (0.85, 1.29)	0.98 (0.77, 1.21)	0.97 (0.72, 1.23)	0.96 (0.59, 1.31)	1.01 (0.76, 1.48)	0.95 (0.72, 1.16)
Nov	1.01 (0.80, 1.26)	1.04 (0.86, 1.29)	1.02 (0.83, 1.27)	1.03 (0.82, 1.34)	1.00 (0.64, 1.31)	1.00 (0.70, 1.32)	1.00 (0.80, 1.24)
Dec	0.92 (0.72, 1.17)	0.94 (0.77, 1.20)	0.87 (0.65, 1.07)	0.94 (0.72, 1.28)	0.93 (0.66, 1.44)	0.92 (0.66, 1.31)	0.88 (0.67, 1.09)

* The intercept refers to a female 79.7 years of age and not hospitalized in the past 30 days in Montreal during January of 2010.

Example Code

```
### Joint modelling of resistance to six antimicrobials in
urinary Escherichia coli isolates in Quebec, Canada ###
### Simulation code for demonstrating Bayesian hierarchical
logistic regression model ###
### Script prepared by Jean-Paul R. Soucy for R 5.3.3 ###

### TO RUN, SAVE THIS SCRIPT AS: Soucy_et_al_model_code_sample.R
###
### ALSO REQUIRED IS THE SUBSEQUENT SCRIPT TO BE IN THE WORKING
DIRECTORY: Soucy_et_al_model_code_sample_fun.R ###

# Load libraries (these packages must be installed with
install.packages() before script can be run)
library(nimble) # mcmc modelling
library(bayesplot) # mcmc posterior plotting
library(ggplot2) # plotting
library(Hmisc) # capitalize

# Load functions (assuming function script is in the working
directory)
source("Soucy_et_al_model_code_sample_fun.R")

# Set random seed (ensure reproducibility of results)
set.seed(as.integer(as.Date("2019-01-01")))

# Part 1: Simulate data

### This section simulates data like that used in the model
underlying the paper.
### First, we randomly generate a dataset of urinary E. coli
isolates.
### We keep the number of observations small so that the models
runs quickly (for illustrative purposes).
### A larger dataset allows for more accurate estimation of model
parameters.
### Next, we generate coefficient values for each of the
covariates for each outcome (type of resistance).
### The coefficients for each covariate are correlated across
outcomes - this gives the data a
### hierarchical stucture.
```

```
### These are the coefficients we are trying to recover using our
modelling strategy.
### Finally, binary outcomes (resistant or not) are generated for
each outcome (type of resistance)
### based on predicted probabilities of resistance from
coefficient values.

## Set number of observations to generate
N <- 1000 # model runs much faster with fewer observations

## Set names of outcomes (resistance to particular antibiotics)
resistance_types <- c("ampicillin", "ciprofloxacin",
"gentamicin",
                      "nitrofurantoin", "tobramycin",
"trimethoprim.sulfamethoxazole")

## Simulate data (e.g., community-acquired urinary E. coli
isolates)

### Generate covariates
dat <- data.frame(
  intercept = 1,
  year = sample(2010:2017, N, replace = TRUE),
  month = sample(1:12, N, replace = TRUE),
  comm_id = sample(0:2, N, replace = TRUE), # community ID
  sex_id = sample(0:1, N, replace = TRUE), # female or male
  hospitalized_past_30_days = sample(0:1, N, replace = TRUE), #
was patient hospitalized in past 30 days
  age_10 = sample(18:95/10, N, replace = TRUE) # age divided by
10
)

### Centre age and square (allows non-linear age effects)
dat$age_10 <- as.numeric(dat$age_10 - mean(dat$age_10))
dat$age_10_square <- as.numeric(dat$age_10^2)

### Create dummy variables for year, month, and community (needed
for modelling)
dat <- dummy_vars(dat) # 2010, January, comm_id = 0 are the
reference groups

## Define constants
```

```
J <- as.integer(length(unique(dat$comm_id)) - 1) # number
community dummies
E <- as.integer(length(unique(dat$year)) - 1) # number of year
dummies
M <- as.integer(length(unique(dat$month)) - 1) # number of month
dummies
Q <- as.integer(length(resistance_types)) # number of antibiotic
resistance outcomes

## Define covariates in model
covariates <- c("sex_id", "age_10", "age_10_square",
"hospitalized_past_30_days",
                paste0("community_", 1:J), # community dummies
                paste0("year_", 1:E), # year dummies
                paste0("month_", 1:M) # month dummies
                )
P <- as.integer(length(covariates) + 1) # total number of
predictors, including intercept
covariates_plus_intercept <- c("intercept", covariates)

## Generate log odds ratios for each variable (and the intercept)
and each outcome, hierarchically across outcomes
## These are the coefficient values our model will try to recover
or_matrix <- matrix(0, nrow = P - 1, ncol = Q) # define matrix
(excluding column for intercept)
or_matrix <- t(apply(or_matrix, 1, function(x) rnorm(n = Q, mean
= rnorm(n = 1, mean = 0, sd = 0.5),
                                                     sd = runif(n
= 1, min = 0.2, max = 0.3)))) # generate values
or_matrix <- rbind(rnorm(n = Q, mean = 0, sd = 0.1), or_matrix) #
add column for the intercept
rownames(or_matrix) <- covariates_plus_intercept # names rows
after coefficients
colnames(or_matrix) <- resistance_types # name columns after
outcomes (resistance types)

## Create placeholders for resistance outcomes
dat <- cbind(dat, matrix(0, nrow = N, ncol = Q, dimnames =
list(1:N, resistance_types)))

## Simulate outcomes for each observation and resistance type
for (res in 1:Q) {
```

```
  ### Linear combination of coefficients
  x <- as.matrix(dat[, covariates_plus_intercept]) %*%
or_matrix[, res]

  ### Convert to probability
  x <- exp(x)/(1 + exp(x))

  ### Convert to binary outcome based on probability
  dat[, resistance_types[res]] <- sapply(x, FUN = function(y)
rbinom(n = 1, size = 1, prob = y))

}

## Preview structure of data
head(dat)

# Part 2: Define and run Bayesian hierarchical logistic
regression model

### This section defines and runs the Bayesian hierarchical
logistic regression model.
### First, the model and its inputs (constants, data, initial
values) are defined.
### Then, the model itself is compiled and run.
### This can take a while (up to ten minutes or longer) depending
on the speed of your computer.
### Finally, the results of the model are exported for plotting.

## Define model
code <- nimbleCode({

  # Logistic regression model with N observations and Q outcomes
  for (i in 1:N) {
    for (q in 1:Q) {
      Y[i, q] ~ dbern(Y.pred[i, q])
    }
    logit(Y.pred[i, 1:Q]) <- X[i, 1:P] %*% beta[1:P, 1:Q]
  }

  # Define priors - all betas are hierarchical
  for (p in 1:P) {
    beta_mu[p] ~ dnorm(mean = 0, sd = 100) # normal prior for
hierarchical mean
```

```
    for (q in 1:Q) {
      beta[p, q] ~ dnorm(mean = beta_mu[p], sd = sigma[p])
    }
  }

  for (p in 1:P) {
    sigma.cauchy[p] ~ dt(mu = 0, sigma = 25, df = 1) # half-
Cauchy prior for hierarchical SDs
    sigma[p] <- abs(sigma.cauchy[p])
  }

})

## Define model inputs

### Constants
constants <- list(
  N = N,
  Q = Q,
  P = P
)

### Data (outcomes and covariates)
Y = as.matrix(dat[, resistance_types])
X = as.matrix(dat[, covariates_plus_intercept])
data <- list(
  Y = Y,
  X = X
)

### Initial values
inits <- list(
  beta_mu = rep(0, times = P),
  beta = matrix(rep(0, times = P*Q), ncol = Q),
  sigma.cauchy = rep(1, times = P)
)

## Build and compile model and MCMC

### Define MCMC settings
n_chains <- 1 # number of chains
n_thin <- 1 # thinning interval (no thinning)
n_iter <- 3000 # number of iterations of each chain
```

```
prop_burnin <- 10 # throw out first 1/n thinned samples
n_burnin <- n_iter/(n_thin*prop_burnin)

### Build model
mod <- nimbleModel(code = code, constants = constants, data =
data, inits = inits, check = FALSE)

### Configure MCMC (this will take a while)
mcmc_conf <- configureMCMC(mod, thin = n_thin)
mcmc_conf$resetMonitors() # manually define monitered parameters
mcmc_conf$addMonitors(c("beta_mu")) # monitor hierarchical means
mcmc_conf$addMonitors("beta") # monitor beta coefficients
mcmc_conf$addMonitors("sigma") # monitor hierarchical SDs

### Build and compile (this will take a while)
mod_mcmc <- buildMCMC(mcmc_conf, enableWAIC = FALSE)
mod_c <- compileNimble(mod)
mod_mcmc_c <- compileNimble(mod_mcmc, project = mod)

### Run MCMC (this will take a while, depending on n_iter)
mod_mcmc_c$run(n_iter, reset = FALSE) # reset = FALSE allows you
to continue running additional iterations later

## Export MCMC samples (results of the model)

### Export samples
samples <- as.matrix(mod_mcmc_c$mvSamples)

### Process samples (rename variables and remove burn-in)
samples <- proc_samples(samples, n_burnin, P, Q, J, E, M,
                        resistance_types, covariates,
covariates_plus_intercept)

### Note: In reality, the model should be run for many more
iterations (i.e., n_iter should be much higher).
### This is because the model does not converge after only 3,000
runs.
### For illustrative purposes, we have kept the number of
iterations shorter than required (faster run).

# Part 3: Plot results

### This section plots the results of our model.
```

```
### Credible intervals will be large since the model has not been
run for sufficiently long.
### First, we plot the estimated ORs for all variables,
individually for each outcome.
### Then, we plot the estimated coefficients for sex_id across
all outcomes, along with the
### esimated hierarchical mean.

## Summarize (medians and 95% credible intervals) odds ratios
(ORs) for each covariate and outcome
or_summary <- posterior_summary(samples, OR = TRUE)
head(or_summary) # preview estimated ORs

## Plot medians and 95% credible intervals for all coefficients
on the OR scale for each outcome (type of resistance)
for (q in 1:Q) {
  samples_temp <- samples[, grep(paste0(", ",
resistance_types[q], "\\]"), colnames(samples))]
  colnames(samples_temp) <- covariates_plus_intercept_names #
rename covariates
  plot(
    mcmc_intervals(exp(samples_temp), point_est = "median", prob
= 0.5, prob_outer = 0.95) +
    ggplot2::labs(title = resistance_types_names[q],
                  x = "OR") + theme(plot.title =
element_text(hjust = 0.5)) # add labels
  )
}

## Plot medians and 95% credible intervals for sex_id
coefficients on the OR scale for all outcomes

### Plot
samples_temp <- samples[, grep("beta_mu\\[sex_id|beta\\[sex_id",
colnames(samples))]
samples_temp <- samples_temp[, c(dim(samples_temp)[2], 1:Q)] #
order hierarchical mean first
colnames(samples_temp) <- c("Hierarchical Mean",
resistance_types_names)
mcmc_intervals(exp(samples_temp), point_est = "median", prob =
0.5, prob_outer = 0.95) +
  ggplot2::labs(title = "Regression coefficients for sex_id",
```

```
                x = "OR") + theme(plot.title = element_text(hjust
= 0.5)) # add labels

### Compare the resistance-specific coefficients from the
previous plot
### to the true values of the coefficients we used to generate
the data
exp(or_matrix["sex_id", ]) # OR scale

### The estimates tend to be in the right neighbourhood (with the
exception of ampicillin),
### but more iterations are required for the model to converge on
the true values.
### Generating a larger sample size (increasing N) would also
help.
### We kept the sample size and number of iterations small to
provide a quicker illustration
### of the model.

### END OF SCRIPT ###
```

```
### Joint modelling of resistance to six antimicrobials in
urinary Escherichia coli isolates in Quebec, Canada ###
### Simulation code for demonstrating Bayesian hierarchical
logistic regression model - Supporting functions ###
### To be loaded by the parent script using source() ###
### Script prepared by Jean-Paul R. Soucy for R 5.3.3 ###

### TO RUN, SAVE THIS SCRIPT AS:
Soucy_et_al_model_code_sample_fun.R ###
### SAVE IN THE WORKING DIRECTORY OF R FOR USE IN THE PARENT
SCRIPT: Soucy_et_al_model_code_sample.R ###

# Function: Convert year, month, and commmunity to dummy
variables for modelling

dummy_vars <- function(dat) {

## Dummary variables for year and month (j - 1 categories per
variable)
year_ <- factor(dat$year - min(dat$year)) # so that dummy
variables start at 1
month_ <- factor(dat$month - 1)
dat <- cbind(dat, model.matrix(~ year_)[, -1])
dat <- cbind(dat, model.matrix(~ month_)[, -1])

## Dummy variables for commmunity
community_ <- factor(dat$comm_id)
dat <- cbind(dat, model.matrix(~ community_)[, -1])

## Convert dummy variables to integer
dat[, grep("^(year_|month_|community_)", names(dat))] <-
  lapply(dat[, grep("^(year_|month_|community_)", names(dat))],
as.integer)

## Return formatted data frame
dat

}

# Function: Summarize coefficient medians and 95% credible
intervals from posterior

posterior_summary <- function(samples, OR = FALSE) {
```

```
  ## Convert to OR scale
  if (OR == TRUE) {
    samples <- exp(samples)
  }

  ## Create summary table
  tab <- as.data.frame(cbind(
    apply(samples, 2, mean),
    apply(samples, 2, median),
    apply(samples, 2, sd),
    apply(samples, 2, function(x) quantile(x, 0.025)),
    apply(samples, 2, function(x) quantile(x, 0.975))))
  names(tab) <- c("mean", "median", "sd", "quant025", "quant975")

  ## Return summary table
  tab

}

# Function: Process and re-name variables in MCMC model to
interpretable names
proc_samples <- function(samples, n_burnin, P, Q, J, E, M,
                         resistance_types, covariates,
covariates_plus_intercept) {

  ## Remove burn-in
  samples <- samples[(n_burnin + 2):nrow(samples), ] # first row
is an extra row of NAs, remove this
  betas_id <- grep("beta\\[", colnames(samples)) # get order of
beta coefficients

  ## Rename betas
  for (p in 1:P) {
    string <- colnames(samples)[grep(paste0("\\[", p, ",|\\[", p,
"\\]"), colnames(samples))]
    colnames(samples)[grep(paste0("\\[", p, ",|\\[", p, "\\]"),
colnames(samples))] <- gsub(paste0("\\[", p), paste0("\\[",
covariates_plus_intercept[p]), string)
  }
  for (q in 1:Q) {
    string <- colnames(samples)[grep(paste0(", ", q, "\\]"),
colnames(samples))]
```

```
    colnames(samples)[grep(paste0(", ", q, "\\]"),
colnames(samples))] <- gsub(paste0(q, "\\]"),
paste0(resistance_types[q], "\\]"), string)
  }

  ## Covariate names to rename for plotting (dummy variables
only)
  covariates_names <- covariates
  covariates_plus_intercept_names <- covariates_plus_intercept

  ## Rename communities, years, and months
  community_names <- c("Quebec", "Rimouski")
  for (j in 1:J) {
    if (length(community_names) != J) stop("Community names are
the wrong length.")
    string_samples <- colnames(samples)[grep(paste0("community_",
j), colnames(samples))]
    string_cov <- covariates_names[grep(paste0("community_", j),
covariates_names)]
    string_cov_int <-
covariates_plus_intercept_names[grep(paste0("community_", j),
covariates_plus_intercept_names)]
    colnames(samples)[grep(paste0("community_", j),
colnames(samples))] <- gsub(paste0("community_", j),
community_names[j], string_samples)
    covariates_names[grep(paste0("community_", j),
covariates_names)] <- gsub(paste0("community_", j),
community_names[j], string_cov)
    covariates_plus_intercept_names[grep(paste0("community_", j),
covariates_plus_intercept_names)] <- gsub(paste0("community_",
j), community_names[j], string_cov_int)
  }
  year_names <- sort(unique(dat$year))[-1]
  for (e in 1:E) {
    string_samples <- colnames(samples)[grep(paste0("year_", e),
colnames(samples))]
    string_cov <- covariates_names[grep(paste0("year_", e),
covariates_names)]
    string_cov_int <-
covariates_plus_intercept_names[grep(paste0("year_", e),
covariates_plus_intercept_names)]
```

```
    colnames(samples)[grep(paste0("year_", e),
colnames(samples))] <- gsub(paste0("year_", e), year_names[e],
string_samples)
    covariates_names[grep(paste0("year_", e), covariates_names)]
<- gsub(paste0("year_", e), year_names[e], string_cov)
    covariates_plus_intercept_names[grep(paste0("year_", e),
covariates_plus_intercept_names)] <- gsub(paste0("year_", e),
year_names[e], string_cov_int)
  }
  month_names <- month.abb[-1] # Do months 10 and 11 first to
avoid issues (matching to 1)
  for (m in 10:M) {
    string_samples <- colnames(samples)[grep(paste0("month_", m),
colnames(samples))]
    string_cov <- covariates_names[grep(paste0("month_", m),
covariates_names)]
    string_cov_int <-
covariates_plus_intercept_names[grep(paste0("month_", m),
covariates_plus_intercept_names)]
    colnames(samples)[grep(paste0("month_", m),
colnames(samples))] <- gsub(paste0("month_", m), month_names[m],
string_samples)
    covariates_names[grep(paste0("month_", m), covariates_names)]
<- gsub(paste0("month_", m), month_names[m], string_cov)
    covariates_plus_intercept_names[grep(paste0("month_", m),
covariates_plus_intercept_names)] <- gsub(paste0("month_", m),
month_names[m], string_cov_int)
  }
  for (m in 1:(M-2)) {
    string_samples <- colnames(samples)[grep(paste0("month_", m),
colnames(samples))]
    string_cov <- covariates_names[grep(paste0("month_", m),
covariates_names)]
    string_cov_int <-
covariates_plus_intercept_names[grep(paste0("month_", m),
covariates_plus_intercept_names)]
    colnames(samples)[grep(paste0("month_", m),
colnames(samples))] <- gsub(paste0("month_", m), month_names[m],
string_samples)
    covariates_names[grep(paste0("month_", m), covariates_names)]
<- gsub(paste0("month_", m), month_names[m], string_cov)
```

```
    covariates_plus_intercept_names[grep(paste0("month_", m),
covariates_plus_intercept_names)] <- gsub(paste0("month_", m),
month_names[m], string_cov_int)
  }

  ## Resistance type names for plotting
  resistance_types_names <- capitalize(gsub("\\.", "/",
resistance_types))
  resistance_types_names[6] <- "TMP/SMX" # name is too long
otherwise

  ## Return names
  assign("resistance_types_names", resistance_types_names, envir
= globalenv())
  assign("covariates_names", covariates_names, envir =
globalenv())
  assign("covariates_plus_intercept_names",
covariates_plus_intercept_names, envir = globalenv())

  ## Return samples
  samples

}

### END OF SCRIPT ###
```
